# Atopic Dermatitis Leading to Blindness: A Frequently Forgotten Sequelae

**DOI:** 10.7759/cureus.60660

**Published:** 2024-05-20

**Authors:** Mohd Khairrudin M Mohd Sobri, Mae-Lynn Catherine Bastion, Chenshen Lam, Mushawiahti Mustapha

**Affiliations:** 1 Opthalmology, Hospital Universiti Kebangsaan Malaysia, Kuala Lumpur, MYS; 2 Ophthalmology, Universiti Kebangsaan Malaysia Medical Centre, Kuala Lumpur, MYS; 3 Ophthalmology, Hospital Universiti Kebangsaan Malaysia, Kuala Lumpur, MYS; 4 Ophthalmology, Hospital Ampang Puteri KPJ, Kuala Lumpur, MYS

**Keywords:** eczema, pars plana vitrectomy, retinal dialysis, retinal detachment, atopic dermatitis

## Abstract

A 12-year-old boy with underlying severeand poorly controlled atopic dermatitis (AD) associated with atopic conjunctivitis and rhinitis presented with a right painless blurring of vision for two weeks. On examination, his right eye visual acuity was 1/60,with grade 1 relative afferent pupillary defect (RAPD). Anterior segment examination revealed anterior uveitis with dense posterior subcapsular cataract and hazy fundus view. B-scan ultrasound suggested a shallow total retinal detachment. Intraoperatively, a large retinal dialysis was found. This paper highlights the need for a high index of suspicion of retinal dialysis in a child with underlying AD and the importance of good control of this systemic condition to prevent ocular morbidity.

## Introduction

Atopic dermatitis (AD) is a chronic and relapsing disease. This skin condition is commonly associated with allergic rhinitis, bronchial asthma, and conjunctivitis. When this eczema becomes severe, a person has patches of skin that are erythematous, swollen, and unbearably itchy, and predisposes the patient to rub the itchy areas [1]. AD can cause ocular complications such as blepharitis, keratoconjunctivitis, keratoconus, glaucoma, cataracts, and rhegmatogenous retinal detachment (RD) [2]. The etiology for each ocular complication in the context of AD and atopy conjunctivitis is complex and multifactorial such as intrinsic immune dysregulation, physical trauma from eye rubbing, and side effects of the medication [2].

Retinal dialysis is a tear in the retina whose anterior edge is at the ora serrata, and the posterior edge is attached to the vitreous base. This is unlike a retinal tear in which the vitreous is attached to the anterior edge of the tear. The most common cause of retinal dialysis is ocular trauma. Historically, there is an established relationship between AD, RD, and retinal dialysis [3]. Although there is an established relationship, the severe sequelae are often forgotten, leading to poor visual outcomes. This case report highlights the importance of a high index of suspicion in managing a pediatric patient with poorly controlled atopy, who had experienced a recent drop in vision [4].

## Case presentation

A 12-year-old boy with underlying severe eczema since 6 months of age and allergic rhinitis presented to an ophthalmologist with right eye blurring of vision for two weeks, preceded by floaters for one month. He described the right eye blurring of vision as generalized and gradually worsening. He denied any history of eye trauma or surgery before this presentation. He had moderate myopia and was wearing spectacles. He had a known case of severe eczema, which was not on proper follow-up, and he had a history of prolonged use of topical steroid cream for his skin condition. He denied previous eye redness, pruritus, or discharge and was not on any regular eye medication. He also denied frequent eye rubbing.

**Figure 1:**
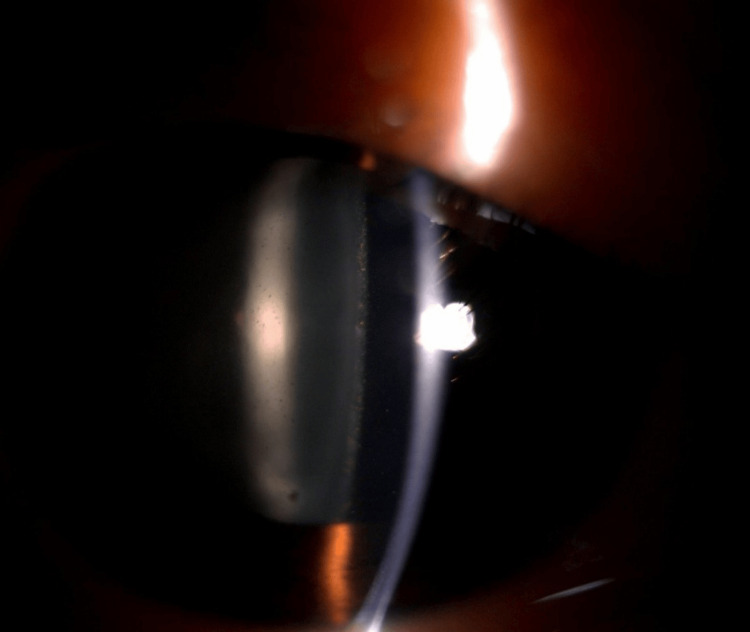
Anterior segment slit beam photograph of the right eye showing anterior chamber cells.

On presentation, his best corrected visual acuity for the right eye was 1/60, and for the left eye, it was 6/9. A right eye grade 1 relative afferent pupillary defect was detected. His anterior segment examination showed multiple small papillae over the upper eyelid, with white conjunctiva in both eyes. Although there were no keratic precipitates, the anterior chamber was deep with 3+ white and pigmented cells in the right eye (Figure 1). The intraocular pressure (IOP) for the right eye was 8 mmHg, and for the left eye, it was 12 mmHg. The right eye fundus view was hazy due to cataracts and vitreous opacities. The left eye anterior segment was otherwise normal, and the fundus view showed a tilted optic disc with a normal macula.

A B-scan of the right eye showed vitreous opacity suggestive of vitritis, and shallow RD inferiorly. The patient was investigated and treated for right eye panuveitis and planned lens aspiration for his right eye cataract first. This decision was made because the inferotemporal RD was shallow, and the cataract did not allow a good fundus view. The B-scan examination also showed vitreous opacity suggestive of vitritis, and we wanted to take a vitreous biopsy first to rule out the cause of panuveitis. Two weeks after presentation, the patient underwent right eye lens aspiration/intraocular lens (IOL) implantation and vitreous biopsy. All investigations sent were negative.

**Figure 2:**
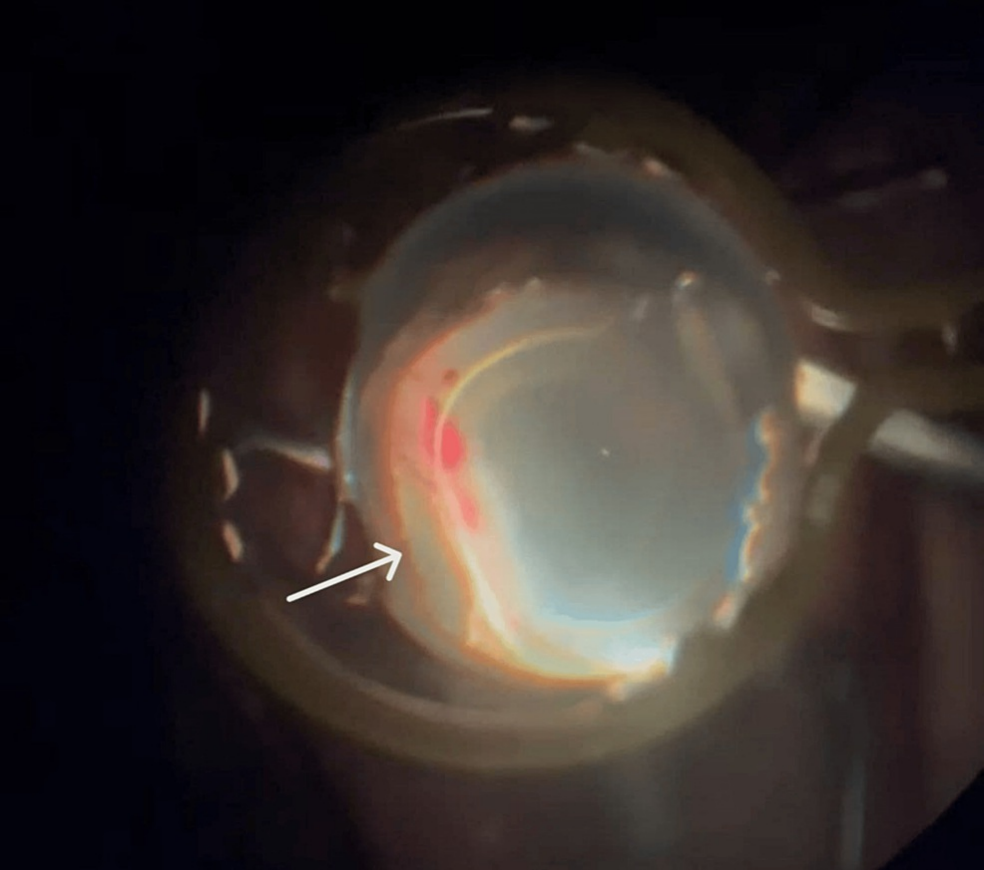
Intraoperative findings viewed through the BIOM fundus viewing system showing anteriorly placed retinal dialysis as the causative break for the RRD. RRD, rhegmatogenous retinal detachment

On day 2 post-op, the fundus was viewed and a bullous RD was noted with Grade A proliferative vitreoretinopathy (PVR). However, there was no visible retinal break. Hence, the patient underwent right scleral buckle/25G pars planar vitrectomy/retinotomy/endo laser/cryopexy/densiron oil surgery under GA, during which retinal dialysis from 4-6 o’clock with total rhegmatogenous RD was seen (Figure 2). The decision to place a scleral buckle intraoperatively was made because of the anterior location of the retinal dialysis. This case was co-managed with a dermatologist, and the patient was started with a tablet of prednisolone 15 mg OD tapering dose and a tablet of azathioprine 50 mg OD for the systemic atopy. Despite good systemic control of eczema with medication, his vision remained poor four months postoperatively, with right eye visual acuity of 2/60, and pH of 2/60. This patient underwent right eye removal of densiron five months after the initial vitrectomy. The retina was attached post-oil removal.

## Discussion

AD is a known chronic disease with a complex etiology. It affects 15% to 20% of children and 1% to 3% of adults in developed countries. Local rates of AD in Malaysia are 13.4% [5]. Allergic conjunctivitis is one of the most common ocular diseases in the pediatric population. Complications with adequate topical treatments are rare. However, treatment is necessary until the immune system reaches maturity, usually around puberty. Till then treatment must be continued so that permanent complications are not encountered. AD is not isolated to skin disease only and has extracutaneous complications. Numerous reports in the literature describe the ocular complications of AD. One of the devastating ocular complications is RD, which can happen in young patients. More severe forms of AD increase the risk of patients getting RDs. A higher incidence of RD in AD patients compared to the general population with RD is 3.22 times more in the AD group [2,6]. This is traced back to the tendency of this population to inadvertently rub their eyes to relieve ocular pruritus. Frequent and aggressive eye rubbing in this patient could have resulted in the RD despite the patient denying it.

Several theories have been postulated as the cause of AD-related RD. One of the theories is the act of frequent eye rubbing or repeated slapping of the eyelid, which is a form of ocular trauma. This can induce mechanical forces to be exerted on the vitreous base resulting in retinal tears anteriorly. It is also suggested that RD in AD may be due to vitreous abnormalities related to ocular immune response to allergy [4,7-9].

Our patient's symptoms and signs were initially attributed to acute panuveitis and treated with topical steroid eyedrops and topical mydriatics, with limited response. The patient also had visually significant cataracts and vitritis, which made posterior segment examination difficult, and a B-scan showed a shallow RD. In AD, a patient can present with rapidly progressive cataracts, as shown in our patient. It is postulated that AD may have a dysfunctional immune response to specific neuroectodermal-derived antigens, such as retinal pigment epithelium (RPE) antigens liberated during retinal tears. Subsequently, the intraocular inflammation caused may mask the presence of RD [9,10]. Prompt surgical intervention might be delayed due to these conflicting signs, to complete the investigation for intraocular inflammation. The shallow RD that occurs in AD is due to a solid vitreous gel encountered in younger age groups of patients. The retinal tears are often located around the ora serrata, and oral dialysis is common due to the inflammation involving the peripheral retina [10,11]. Our patient also developed low IOP, which is explained by the increased outflow of aqueous through the opened subretinal space or decreased aqueous production from depressed ciliary function resulting from inflammation or ciliary detachment [11].

Upon diagnosis, the surgical approach has to be decided properly as RD in AD in younger age groups tends to develop severe PVR and a higher rate of RD recurrence post-surgical intervention. A combined scleral buckle and vitrectomy is beneficial because there is a relatively higher incidence of retinal re-detachment that can occur anteriorly, and there is also an increased incidence of anterior proliferative vitreoretinopathy in AD-related RD due to poor vitreous clearance in the pediatric vitreous. The scleral buckle also has the potential to reduce the risk of retinal dialysis recurrence. Early diagnosis is essential so that early surgical intervention can be carried out before PVR develops and saves the patient's vision [12,13].

## Conclusions

Our case shows that a high index of suspicion of RD and retinal dialysis is needed when a patient with underlying dermatitis presents with cataracts and panuveitis so that surgical intervention is not delayed. Dermatologists and ophthalmologists should co-manage patients with severe dermatitis requiring prolonged steroid or second-line agent use so that every patient’s disease can be adequately controlled and these ocular complications can be prevented or adequately addressed on time.
